# A Systems Biology Approach Using Transcriptomic Data Reveals Genes and Pathways in Porcine Skeletal Muscle Affected by Dietary Lysine

**DOI:** 10.3390/ijms18040885

**Published:** 2017-04-21

**Authors:** Taiji Wang, Jean M. Feugang, Mark A. Crenshaw, Naresh Regmi, John R. Blanton, Shengfa F. Liao

**Affiliations:** Department of Animal and Dairy Sciences, Mississippi State University, Starkville, MS 39762, USA; wangtaiji@hotmail.com (T.W.); jn181@msstate.edu (J.M.F.); mac4@msstate.edu (M.A.C.); regminar@msu.edu (N.R.); jblanton@ads.msstate.edu (J.R.B.)

**Keywords:** lysine, skeletal muscle, transcriptome, gene expression, microarray, pig

## Abstract

Nine crossbred finishing barrows (body weight 94.4 ± 6.7 kg) randomly assigned to three dietary treatments were used to investigate the effects of dietary lysine on muscle growth related metabolic and signaling pathways. Muscle samples were collected from the longissimus dorsi of individual pigs after feeding the lysine-deficient (4.30 g/kg), lysine-adequate (7.10 g/kg), or lysine-excess (9.80 g/kg) diet for five weeks, and the total RNA was extracted afterwards. Affymetrix Porcine Gene 1.0 ST Array was used to quantify the expression levels of 19,211 genes. Statistical ANOVA analysis of the microarray data showed that 674 transcripts were differentially expressed (at *p ≤* 0.05 level); 60 out of 131 transcripts (at *p* ≤ 0.01 level) were annotated in the NetAffx database. Ingenuity pathway analysis showed that dietary lysine deficiency may lead to: (1) increased muscle protein degradation via the ubiquitination pathway as indicated by the up-regulated *DNAJA1*, *HSP90AB1* and *UBE2B* mRNA; (2) reduced muscle protein synthesis via the up-regulated *RND3* and *ZIC1* mRNA; (3) increased serine and glycine synthesis via the up-regulated *PHGDH* and *PSPH* mRNA; and (4) increased lipid accumulation via the up-regulated *ME1*, *SCD*, and *CIDEC* mRNA. Dietary lysine excess may lead to: (1) decreased muscle protein degradation via the down-regulated *DNAJA1*, *HSP90AA1*, *HSPH1*, and *UBE2D3* mRNA; and (2) reduced lipid biosynthesis via the down-regulated *CFD* and *ME1* mRNA. Collectively, dietary lysine may function as a signaling molecule to regulate protein turnover and lipid metabolism in the skeletal muscle of finishing pigs.

## 1. Introduction

The growth and development of skeletal muscle of pigs essentially requires dietary supply of proteins, or amino acids (AAs), to be exact. Of the 20 AAs that serve as building blocks for protein biosynthesis, lysine makes up the biggest part of the body protein gain in growing-finishing pigs (7.1 g lysine per 100 g body protein) [1], but lysine typically is also the first limiting AA in conventional grain based swine diets [1,2]. In the meantime, lysine is a truly essential AA for pig’s life because it cannot be de novo synthesized from any other nutrients or nutrient metabolites within pig’s body. Therefore, dietary inclusion of sufficient lysine is necessary to optimize pig’s growth performance.

It has been reported that dietary lysine supplementation can improve pig’s growth performance, especially the retention of nitrogen [3,4]. Dietary deficiency of lysine can affect pig carcass characteristics by decreasing the lean meat percentage [5], increasing the subcutaneous fat depth [5,6], and increasing the intramuscular fat content of *longissimus dorsi* muscle [5,6,7]. However, the underlying molecular mechanisms by which dietary lysine directly or indirectly regulates muscle protein accretion and fat deposition in pigs are still not clear.

Recent research advance revealed a key role AAs play as nutritional signals in regulation of cell signaling processes [8]. For example, leucine has been found to activate the mammalian target of rapamycin (mTOR) pathway to stimulate the initiation of mRNA translation in skeletal muscle of mammals [9]. Similarly, dietary arginine supplementation increased the activity of mTOR signaling in skeletal muscle of neonatal pigs [10]. Glutamine may prevent protein hypercatabolism via inhibition of myostatin hyperexpression [11], and supplementation of glutamine to a high-fat diet improved insulin signaling in the muscle of rats [12]. In terms of lysine, however, which signaling pathways related to muscle protein accretion and carcass characteristics are regulated by its dietary supply is still unknown.

To greatly improve the feed efficiency of raising pigs to meet the increasing demands for more high-quality pork products worldwide, further understanding of the molecular mechanisms by which dietary AAs regulate lean muscle growth is urgently needed. Therefore, the objective of this study is to explore the effect of dietary lysine on the metabolic and cell signaling pathways associated with the growth performance, especially, the muscle growth, of finishing pigs.

## 2. Results

The effects of dietary lysine at three different levels on the market-value associated carcass characteristics, along with some growth performance data, generated for this project were also the scientific interest of this research group and have been reported by Wang et al. [13]. This present report focuses on the effects of dietary lysine on skeletal muscle gene expression at the transcriptomic level.

### 2.1. Bioinformatics Analyses of Microarray Data

All the microarray *.cel files generated from the GeneChip Operating Software (GCOS; Affymetrix, Inc., Santa Clara, CA, USA) as well as the GC Robust Multichip Averaging-corrected data processed with the Partek Genomics Suite (PGS) software (version 6.6; Partek Inc., St. Louis, MO, USA) have been deposited in the database of Gene Expression Omnibus (GEO) at the National Center for Biotechnology Information (http://www.ncbi.nlm.nih.gov/geo) following the standards of minimum information about a microarray experiment (MIAMIE) compliant [14]. The series accession number for the deposition assigned by the GEO is GSE77708.

**Figure 1:**
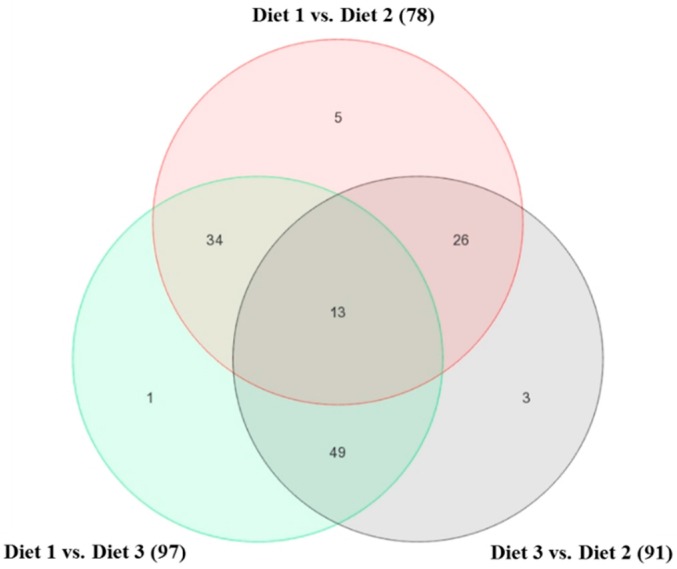
Venn diagram detailing the number of differentially expressed genes or gene transcripts (DEG; *p ≤* 0.01) in the skeletal muscle of finishing pigs fed three different diets. The number of DEG between each two dietary treatments is shown in parentheses.

**Figure 2:**
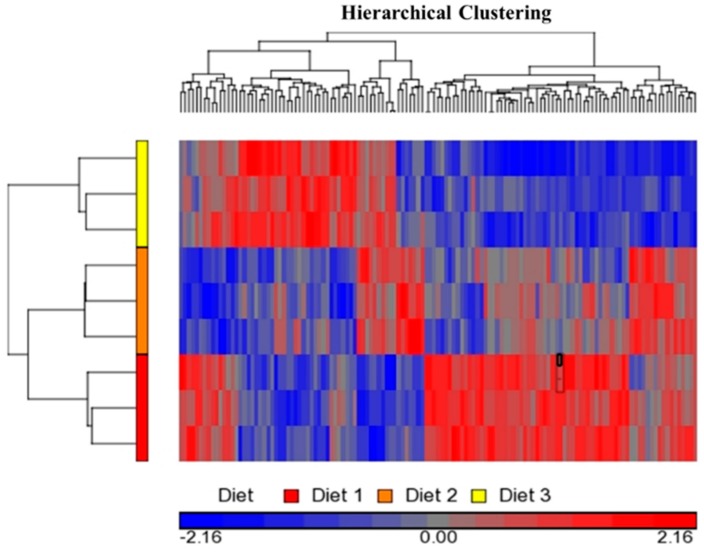
Hierarchical clusters of differentially expressed genes or gene transcripts (DEG; *p ≤* 0.01). The expression level for each gene transcript was standardized to mean of 0 and scale to Standard Deviation (SD) of 1, which is the default setting of the Partek Genomics Suite software (Partek Inc., St. Louis, MO, USA). As indicated by the legend color box, gray color in the middle represents the mean value, 0, red color represents gene expression level above the mean, and blue color below the mean. The intensity of the color reflects the relative intensity of the fold change.

The raw mRNA abundance values (i.e., the *.cel files) were imported into the PGS for ANOVA statistical analysis, which showed 674 transcripts differentially expressed (*p ≤* 0.05) in the *longissimus dorsi* muscle of the pigs fed different diets. To refine this analysis, 131 transcripts with *p ≤* 0.01 were considered as differentially expressed genes or gene transcripts (DEG) (Table S1). The overall dietary treatment effect on these 131 DEG is shown on Figure 1. Hierarchical cluster analysis of these DEG using PGS software showed that associated with the three dietary treatments are three clusters that have three distinct gene expression patterns (Figure 2). Of these 131 transcripts, 60 were annotated and 71 were unannotated in the NetAffx annotation database (Affymetrix, Inc.), and the 60 annotated transcripts belong to 59 genes.

**Figure 3:**
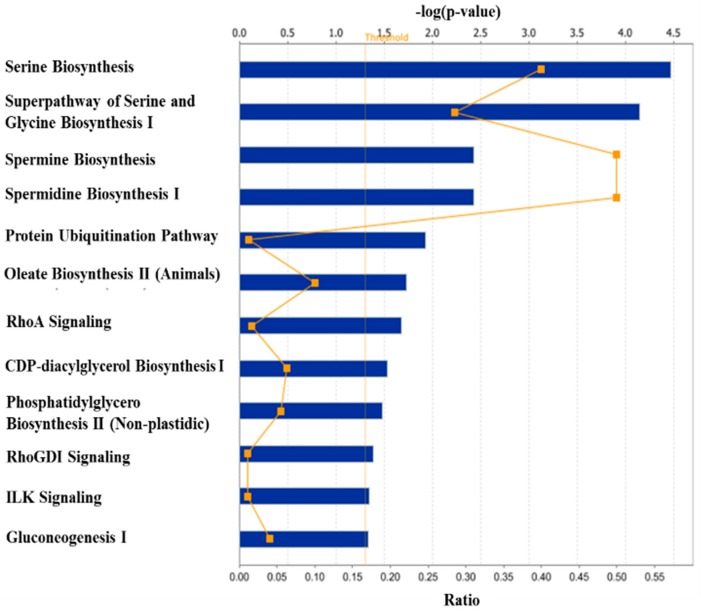
Canonical pathways significantly affected (a “−log of *p*-value” ≥1.3) by dietary lysine deficiency in skeletal muscle of finishing pigs. The *x*-axis displays the significant pathways, and the *y*-axis displays the −log of *p*-value. Each rectangular symbol connected with other rectangular symbols by a line represents a ratio that is calculated as the number of genes in a given pathway that meet the defined cutoff criteria divided by the total number of genes in the reference gene set that make up that pathway.

The orthogonal contrast analysis of the dietary treatments revealed a total of 40 genes differentially expressed in the skeletal muscle of the pigs fed Diet 1 vs. the pigs fed Diet 2, of which 28 genes were up-regulated and 11 were down-regulated (Table 1). Canonical pathway analysis of these 40 genes with the online Ingenuity Pathways Analysis (IPA) software showed that several cell signaling and metabolic pathways in skeletal muscle could have been affected (*p ≤* 0.05) by dietary deficiency of lysine (Figure 3), of which the Top 5 are responsible for the serine and glycine biosynthesis pathways, protein ubiquitination pathway, and spermine and spermidine biosynthesis pathways.

**Figure 4:**
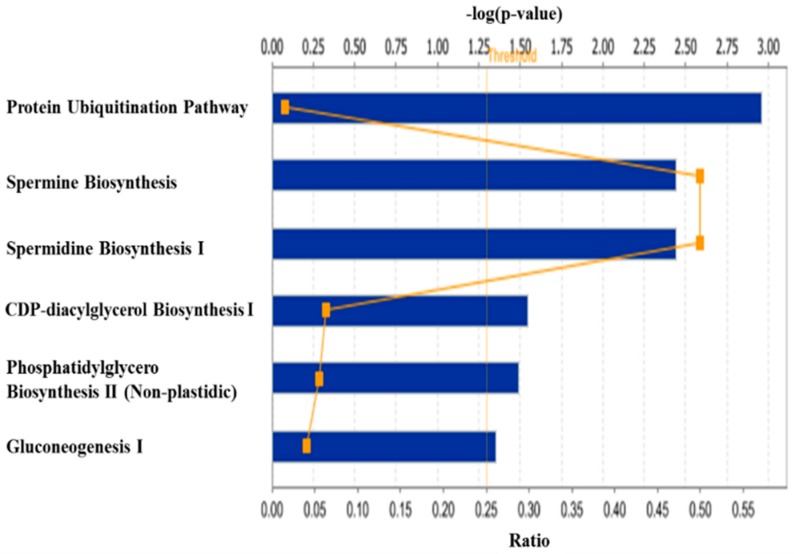
Canonical pathways significantly affected (a “−log of *p*-value” ≥ 1.3) by dietary lysine excess in skeletal muscle of finishing pigs. The *x*-axis displays the significant pathways, and the *y*-axis displays the −log of *p*-value. Each rectangular symbol connected with other rectangular symbols by a line represents a ratio that is calculated as the number of genes in a given pathway that meet the defined cutoff criteria divided by the total number of genes in the reference gene set that make up that pathway.

The orthogonal contrast analysis of the dietary treatments also revealed a total of 35 genes differentially expressed in the skeletal muscle of the pigs fed Diet 3 vs. the pigs fed Diet 2, of which 13 genes were up-regulated and 22 were down-regulated (Table 2). Canonical pathway analysis of these 35 genes with the Core Analysis program of the IPA software showed that several cell signaling and metabolic pathways in the skeletal muscle of pigs might have been affected (*p ≤* 0.05) by dietary excess of lysine (Figure 4), of which the Top 5 are responsible for protein ubiquitination pathway, spermine and spermidine biosynthesis pathways, CDP-diacylglycerol biosynthesis pathway, and phosphatidylglycerol biosynthesis.

### 2.2. Real-Time RT-PCR Analysis of Selected Genes

Nine genes involved in serine and glycine biosynthesis, lipid biosynthesis, spermine and spermidine biosynthesis, and protein ubiquitination pathway, identified from the bioinformatics analyses of the microarray data, were selected for validation using semi-quantitative real-time RT-PCR analysis: *PHGDH* (phosphoglycerate dehydrogenase), *PSPH* (phosphoserine phosphatase), *SCD* (stearoyl-CoA desaturase), *CIDEC* (cell death-inducing DFFA-like effector c), *AMD1* (adenosylmethionine decarboxylase 1), *ZIC1* (Zic family member 1), *ERLEC1* (endoplasmic reticulum lectin 1), *DNAJA1* (DnaJ (Hsp40) homolog subfamily A member 1), and *ZNF181* (Zinc finger protein 181).

**Figure 5:**
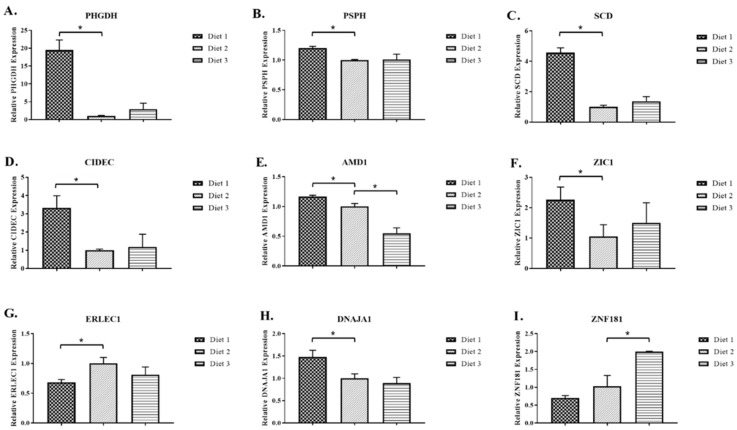
Semi-quantitative real-time RT-PCR analyses of: *PHGDH* (**A**); *PSPH* (**B**); *SCD* (**C**); *CIDEC* (**D**); *AMD1* (**E**); *ZIC1* (**F**); *ERLEC1* (**G**); *DNAJA1* (**H**); and *ZNF181* (**I**) mRNA expression in the skeletal muscle of pigs fed a lysine-deficient diet (Diet 1), a lysine-adequate diet (Diet 2), and a lysine-excess diet (Diet 3). The *y*-axis shows the relative mRNA expression levels relative to the D2 pig group. The error bars denote SD, and the * signs denote differential expressions (*p ≤* 0.05). *PHGDH* = Phosphoglycerate dehydrogenase; *PSPH* = Phosphoserine phosphatase; *SCD* = Stearoyl-CoA desaturase; *CIDEC* = Cell death-inducing DFFA-like effector c; *AMD1* = Adenosylmethionine decarboxylase 1; *ZIC1* = Zic family member 1; *ERLEC1* = Endoplasmic reticulum lectin 1; *DNAJA1* = DnaJ (Hsp40) homolog, subfamily A, member 1; *ZNF181* = Zinc finger protein 181.

As shown in Figure 5, the levels of *PHGDH*, *PSPH*, *SCD*, *CIDEC* and *ZIC1* mRNA expressed in the muscle of the pigs fed Diet 1 were significantly higher (*p ≤* 0.05) than those in the pigs fed Diet 2, and there were no differences in the mRNA levels of these five genes between the pigs fed Diet 3 vs. Diet 2 (Figure 5A–D,F). The level of the *AMD1* mRNA expressed in the muscle of the pigs fed Diet 1 was higher (*p ≤* 0.05) than that in the pigs fed Diet 2, while its level in the pigs fed Diet 3 was lower (*p ≤* 0.05) than that in the pigs fed Diet 2 (Figure 5E). All of these results are consistent with the results obtained from the microarray analysis concerning these genes (Table 1 and Table 2).

The level of *ERLEC1* mRNA in the muscle of pigs fed Diet 1 was significantly lower (*p ≤* 0.05) than that of pigs fed Diet 2 (Figure 5G), while the expression level of *DNAJA1* in the muscle of pigs fed Diet 1 was significantly higher (*p ≤* 0.05) than that of pigs fed Diet 2 (Figure 5H). The level of *ZNF181* mRNA in the muscle of pigs fed Diet 3 was significantly higher (*p ≤* 0.05) than that of pigs fed Diet 2 (Figure 5I). These results were also consistent with the results obtained from the microarray analysis concerning these two genes (Table 1). When compared to Diet 2, the expression levels of *ERLEC1* and *DNAJA1* mRNA in the pigs fed Diet 3 and the expression level of *ZNF181* mRNA in the pigs fed Diet 1 were all numerically decreased (Figure 5G–I), which is also in agreement with the results obtained from the microarray analysis concerning these two genes (Table 2).

## 3. Discussion

To the best of our knowledge, this study was the first to explore the transcriptomic profile of skeletal muscle of finishing pigs in response to dietary lysine levels. Approximately 130 genes or gene transcripts involved in different signaling and metabolic pathways were found to be affected by dietary lysine. Of these pathways, the significant ones are those associated with protein and lipid metabolism and muscle growth.

### 3.1. Regulation of Protein Turnover

#### 3.1.1. Protein Degradation

It has been shown that dietary lysine may regulate protein degradation in skeletal muscle of pigs [3]. Ubiquitin-proteasome signaling pathway plays important roles in the breakdown of myobrillar protein in skeletal muscle [15], which involves two successive steps: conjugation of multiple ubiquitin moieties to the target protein and degradation of the polyubiquitinated protein by the 26S proteasome complex. In this study, it was shown that lysine might affect the protein ubiquitination pathway through regulating at least 6 genes, *DNAJA1*, *HSP90AA1*, *HSP90AB1*, *HSPH1*, *UBE2B* and *UBE2D2*. More specifically, when compared to the lysine-adequate pigs (i.e., the Diet 2 group), dietary lysine deficiency increased the mRNA levels of *DNAJA1*, *HSP90AB1*, and *UBE2B* (Table 1), while dietary lysine excess decreased the mRNA levels of *DNAJA1*, *HSP90AA1*, *HSPH1*, and *UBE2D2* (Table 2). That the expression levels of the genes related to protein ubiquitination pathway were down-regulated with the increased level of dietary lysine implies that a high level of dietary lysine may suppress protein degradation and that dietary lysine deficiency may stimulate protein degradation through the ubiquitin-proteasome degradation pathway. This implication is supported by an in vivo study [16], which showed that dietary lysine supplementation inhibited the ubiquitin-proteasome pathway in the skeletal muscle of rats.

Cystatin 9-like (*CST9L*), a cysteine protease inhibitor, prevents or reduces the activity of a cysteine protease (a.k.a. cysteine-type endopeptidase), which is an enzyme that can degrade proteins in animal body. When present at a high level or when abnormally activated, cysteine proteases are thought to be involved in numerous pathophysiological processes, such as muscular dystrophy [17]. In this study, the expression or abundance of *CST9L* mRNA was down-regulated by the lysine-deficient diet (Table 1), which means that the activity of cysteine-type endopeptidase might have been enhanced, leading to an increased protein degradation in skeletal muscle.

The endoplasmic reticulum (ER) lectin 1 (*ERLEC1*) gene encodes XTP3-B lectin, an ER protein that can function as a negative regulator of ER-associated protein degradation (via its interaction with the membrane-associated ubiquitin ligase complex), and can protect newly synthesized immature polypeptides from premature degradation [18]. The level of *ERLEC1* mRNA was down-regulated in skeletal muscle of pigs fed Diet 1 or 3. The connection between the expression level of *ERLEC1* mRNA and protein degradation in skeletal muscle associated with dietary lysine level is not clear and needs further investigation.

Based on our previous report [13], lysine deficiency was shown to significantly decrease the average daily gain (ADG) in finishing pigs, while there is no significant difference in the ADG between the pigs fed the lysine-excess and lysine-adequate diets. Data from this study suggest that the increased muscle protein degradation might be the reason for the decreased ADG when pigs were fed a lysine-deficient diet. However, whether the decreased expression of *DNAJA1*, *HSP90AA1*, *HSPH1*, and *UBE2D2* caused by the excess of dietary lysine (Table 2) can lead to significantly decreased muscle protein degradation warrants further investigation.

It was reported that *DNAJA1* gene encodes Hsp40 (heat shock protein 40), which is a negative marker of meat tenderness [19]. Interestingly, in this study, we found that the level of *DNAJA1* mRNA was decreased along with the increasing levels of dietary lysine (Table 1 and Table 2). Therefore, dietary lysine deficiency may negatively affect pork quality, while dietary lysine excess may improve pork tenderness via the regulation of *DNAJA1* gene expression in skeletal muscle.

#### 3.1.2. Protein Synthesis

Several genes associated with protein synthesis, such as *RND3* (Rho family gtpase 3) and *ZIC1* (Zic family member 1), were regulated by dietary lysine in this study. *RND3* is a member of Rnd family, a sub-group of the Rho family of small GTP-binding proteins. The Rnd family is comprised of three proteins including Rnd1/Rho6, Rnd2/Rho7, and Rnd3/Rho8/RhoE [20]. Upregulation of *RND3* expression inhibited cell proliferation, which is associated with PTEN/PI3K/Akt signaling pathway [21]. *ZIC1* encodes a member of the ZIC family of C2H2-type zinc finger proteins, and is an activator of Wnt signaling [22]. Overexpression of *ZIC1* resulted in inactivation of PI3K and MAPK signaling pathways, which can further regulate multiple downstream targets [23]. The PI3K/Akt signaling pathway has a number of downstream effectors including mTOR, which can affect the transcription of p70 or 4EBP1 and regulate protein synthesis [24].

The results of this study showed that dietary lysine deficiency up-regulated the expression levels of *RND3* and *ZIC1* mRNA, which might lead to inhibition of PI3K/Akt signaling pathway, causing inactivation of downstream effectors of mTOR pathway and resulting in less muscle protein synthesis. Sato et al. [25] reported that lysine could in part affect the Akt pathway in C2C12 murine myotubes, and that the protein synthesis may be increased by lysine through mTOR pathway. The lower ADG, lower lean cut, and reduced ham weight of the pigs fed the lysine-deficient diet reported by Wang et al. [13] may be in part due to the reduced muscle protein synthesis via the up-regulation of *RND3* and *ZIC1* gene expression.

**Figure 6:**
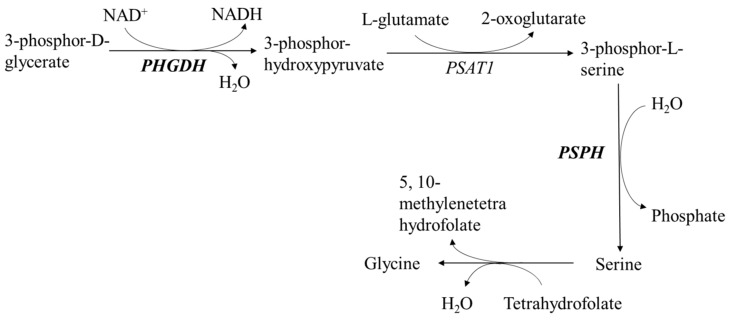
Roles of phosphoglycerate dehydrogenase (*PHGDH*) and phosphoserine phosphatase (*PSPH*) in the serine and glycine biosynthesis pathway (Adapted from Ingenuity Pathways Analysis (IPA, 8.0-2602) online software (http://www.ingenuity.com; Ingenuity Systems, Inc., Redwood City, CA, USA).

The serine and glycine biosynthesis pathway consists of three sequential enzymatic reactions (Figure 6), which begins with the oxidation of glycolytic intermediate phosphoglycerate (3PG) to 3-phosphohydroxypyruvate (pPYR) with the concomitant reduction of NAD^+^ co-factor to NADH by an enzyme called phosphoglycerate dehydrogenase (*PHGDH*). Phosphoserine amino transferase (PSAT1) then uses the α-amino group of glutamate to transaminate pPYR yielding phosphoserine (pSER) and α-ketoglutarate (αKG). Finally, phosphoserine phosphatase (*PSPH*) dephosphorylates pSER to give serine, which will interconvert with glycine in animal body [26]. This study revealed that dietary lysine deficiency increased the expression of *PHGDH* and *PSPH* mRNA, responsible for coding two rate-limiting enzymes in the serine and glycine biosynthesis pathway (Figure 6), and these increases indicated a positive serine and/or glycine biosynthesis in the skeletal muscle of the finishing pigs. One of our previous studies showed that the plasma concentrations of serine (numerically) and glycine (*p ≤* 0.05) were both decreased in the pigs fed a lysine-deficient diet [27], and these decreases suggested that there might not be enough serine and glycine supply in the plasma, causing active serine and glycine biosyntheses in skeletal muscle. Since serine and glycine are building blocks for protein synthesis and substrates for creatine, purine, and pyrimidine generation [28], the change of *PHGDH* and *PSPH* mRNA levels implies that dietary lysine could affect muscle energy metabolism, and the biosyntheses of nuclear acids and proteins.

The biosynthesis pathways for two polyamines, spermine and spermidine, may be affected by dietary lysine through regulating the expression of *AMD1*, which encodes *S*-adenosylmethionine decarboxylase 1 (AdoMetDC), a key enzyme in the rate-limiting step of polyamine biosynthesis [29]. Although the potential mechanism by which polyamines regulate skeletal muscle hypertrophy and atrophy are still unclear, there is a strong association between polyamine levels and muscle mass [29,30]. The *AMD1* mRNA level affected by dietary lysine may lead to a change in the polyamine level causing differential muscle growth. Regmi et al. [27] reported that the plasma concentration of arginine decreased along with the increased dietary lysine concentration. Since arginine is the main substrate for polyamine synthesis [31], the regulation of dietary lysine on *AMD1* expression and then polyamine level may be due to the antagonism effect of lysine on plasma arginine level.

### 3.2. Regulation of Lipid Metabolism

**Figure 7:**
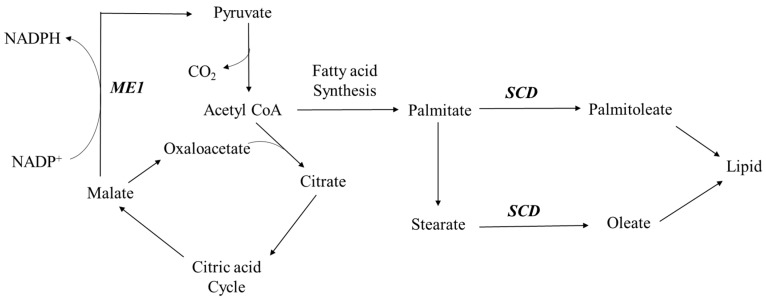
Roles of malic enzyme 1 (ME1) and stearoyl-CoA desaturase (SCD) in lipid biosynthesis pathway.

Several DEG in Table 1 and Table 2, including *ME1* (malic enzyme 1), *SCD* (stearoyl-CoA desaturase), and *CIDEC* (cell death-inducing DFFA-like effector c), are associated with lipid biosynthesis. *ME1* catalyzes the reversible oxidative decarboxylation of L-malate to pyruvate (Figure 7), which links glycolytic pathway and citric acid cycle, involving the reduction of NADP^+^ to NAD(P)H [32]. At the same time, a malic enzyme can form part of the tricarboxylate shuttle, which releases acetyl-CoA from the mitochondria into the cytosol [32]. Both NADPH and acetyl-CoA are substrates of fatty acid biosynthesis, and malic enzyme activity has a strong influence on intramuscular fat content [33]. In addition, a significant association between *ME1* genotype and back-fat thickness of pigs has been reported and it was found that different malic enzyme activities are highly related to the fatness traits of different pig breeds [32]. Therefore, the increased level of *ME1* mRNA in the skeletal muscle of the pigs fed the lysine-deficient diet might be associated with the enhanced lipid biosynthesis. Furthermore, the level of *ME1* mRNA was decreased in pigs fed the lysine-excess diet. It seems that increasing dietary lysine level from deficiency to excess, the level of *ME1* mRNA was decreased accordingly, which implies that ME1 enzyme may be a key regulator of lipid biosynthesis in response to dietary lysine. Specifically, dietary supplementation of lysine may inhibit lipid biosynthesis through the reduction of *ME1* gene expression.

As shown in Figure 7, the end product of fatty acid synthesis from acetyl-CoA is palmitate, which can be elongated to stearate. SCD is the rate-limiting lipogenic enzyme in the biosynthesis of monounsaturated fatty acids [34]. It has been proven that the expression of *SCD* positively correlates with intramuscular fat deposition in pigs [35,36]. *CIDEC* gene is also associated with body lipid accumulation, and elevated *CIDEC* expression resulted in an inhibition of fatty acid oxidation and an increase of de novo lipogenesis in muscle cells [37]. Both *SCD* and *CIDEC* mRNA were up-regulated in the lysine-deficient pigs, which is, at least partially, supported by da Costa et al. [36], who reported that when the dietary crude protein and lysine levels were low, the *SCD* mRNA expression was increased in the skeletal muscle of growing pigs.

The level of *CFD* (complement factor D, a.k.a. adipsin) mRNA in the skeletal muscle of finishing pigs was reduced after the lysine-excess diet was fed for 5 weeks (Table 2). As is known, CFD interacts with complement C3 and factor B to form acylation-stimulating protein, which is involved in stimulating glucose transport, enhancing fatty acid re-esterification, and inhibiting lipolysis [38]. Lan et al. [39] reported that a higher *CFD* level was associated with an increased fat content in skeletal muscle of the obese mice. The decreased *CFD* mRNA level in the lysine-excess group of this study may be associated with the reduced fatty acid synthesis and enhanced lipolysis, leading to a decreased fat deposition in the muscle when compared to the lysine-adequate pig group.

It has been well accepted that dietary lysine deficiency can lead to intramuscular fat accumulation in *longissimus dorsi* [5,6,7]. Our study implies that dietary lysine deficiency may promote lipid accumulation in skeletal muscle via increasing *ME1*, *SCD*, and *CIDEC* mRNA expression. It is interesting to mention that one of our previous studies [40] found that plasma concentration of cholesterol was significantly increased in pigs fed a lysine-deficient diet. As is known, SCD can catalyze the conversion of palmitate and stearate to their corresponding unsaturated fatty acids, which can be further used for the synthesis of cholesterol and phospholipid [34]. The active lipid accumulation in muscle and plasma might be due to lysine deficiency which restricts protein synthesis with surplus energy being converted to lipids. As aforementioned, dietary lysine deficiency stimulated serine synthesis, while serine serves as a substrate for synthesis of two membrane lipid molecules, phosphatidylserine and sphingolipids, which can be efficiently incorporated into cell membrane lipid bilayer [41]. Therefore, the increased serine synthesis in the lysine-deficient group may contribute to lipid biosynthesis as well.

Dietary lysine deficiency decreased the expression of the *ESYT1* (extended synaptotagmin-like protein 1) mRNA, which encodes E-Syt1 (extended synaptotagmins). E-Syt1 are endoplasmic reticulum (ER) proteins that participate in tethering function between the ER and the plasma membrane (PM) and have roles in lipid transport between the two membranes [42]. The connection between ER and PM is dynamically regulated by Ca^2+^ signaling, of which elevation of cytosolic Ca^2+^ triggered translocation of E-Syt1 to ER-PM junctions to enhance ER-to-PM connection [43]. Dietary lysine supplementation has been shown to regulate Ca^2+^ metabolism [44], specifically enhancing intestinal Ca^2+^ absorption and improve the renal conservation of the absorbed Ca^2+^. Therefore, the decreased level of ESYT1 mRNA in the lysine-deficient group of this study may be attributed to the effect of lysine on Ca^2+^ absorption, while at the same time lysine may affect lipid transport through E-Syt1.

Lysine may affect lipid oxidation through regulation of *ALKBH7* (alkB, alkylation repair homolog 7), *LGALS13* (lectin, galactoside-binding, soluble, 13), and *LCLAT1* (lysocardiolipin acyltransferase 1) expression. *ALKBH7* encodes a mitochondrial resident protein, which is located in the mitochondrial matrix and involved in fatty acid metabolism. *ALKBH7* deletion has been shown to dramatically increase body fat and body weight, which may be due to the fact that ALKBH7 directly or indirectly facilitates the utilization of short-chain fatty acids [45]. *LGALS13* encodes protein that has lysophospholipase activity, which was shown to be involved in phospholipid metabolism [46]. Deficiency of *LCLAT1*, which encodes acyl-CoA:lysocardiolipin acyltransferase 1, has been shown to increase lipid oxidation and decrease the content of lipids in mouse [47]. Therefore, lysine may affect lipid content by regulating the expression of *ALKBH7*, *LGALS13*, and *LCLAT1*.

### 3.3. Other Biological Processes

The mRNA levels of several genes related to some other biological processes were also altered by the level of dietary lysine (Table 1 and Table 2). Dietary lysine deficiency may affect the metabolism of macromolecules by increasing the expression of *FUT1* (fucosyltransferase 1) and *CHIT1* (chitinase 1), both of which are involved in the metabolic process of carbohydrates [48]. Dietary lysine deficiency increased the mRNA levels of *TXNL1* (thioredoxin-like 1) and *CD164* (an 80 to 100 kDa type 1 transmembrane sialomucin), encoding proteins involved in cell growth [49], of which TXNL1 may be regulated by Akt-mTOR pathway [50]. Lysine deficiency also decreased the expression of *RASSF7* (Ras association domain family member 7), which is a member of the N-terminal Ras association domain family. RASSF7 negatively regulates pro-apoptotic JNK signaling [51], which is a member of an evolutionarily conserved sub-family of mitogen-activated protein (MAP) kinases and important as both positive and negative modulators of apoptosis [52]. Knocking down of RASSF7 has been shown to inhibit cell growth [53].

Dietary lysine-excess down-regulated the expression of *SLC9A2* (solute carrier family 9, subfamily A, member 2), which is involved in the transport of Na^+^ and contributes to the regulation of intracellular pH [54]. Dietary lysine-excess increased the expression of *CNTROB* (centrobin, centrosomal BRCA2 interacting protein) and *PDCL* (phosducin-like), of which CNTROB protein is involved in centriole duplication [55], and PDCL protein has been shown to regulate G-protein signaling by binding to the β-γ subunits of G proteins [56].

In addition, several other genes related to gene transcription and protein translation in the skeletal muscle were also affected by dietary lysine. The genes encoding the class of transcription factors, called homeobox genes, were found in clusters named A, B, C, and D. *HOXA11* and *HOXA4* are part of the A cluster and encode DNA-binding transcription factors which may regulate gene expression. *HOXA11* expression increases transcription of DNA [57] and was regulated by PTEN [58]. The function of ZNF181 (zinc finger protein 181) may be related to DNA binding and gene regulation [59]. ZMAT5 (zinc finger, matrin-type 5) is involved in mRNA splicing. MORF4L2 (MRGX) is a novel transcription factor and can repress or activate the B-myb promoter [60]. *GABPB1* (GA binding protein transcription factor, β subunit 1) encodes the GA-binding protein transcription factor, β subunit, which forms a tetrameric complex with the α subunit, and influence the DNA binding stability of hGABP α and regulate hGABP-mediated transcription [61].

Translational control plays an essential role in regulating the expression of gene to mRNA and to protein. Members of CPEB (cytoplasm polyadenylation element binding protein) family have been shown to bind to the 3′ UTR of target mRNAs and regulate their translation, of which CPEB2 interacts with the elongation factor, eEF2, to reduce the eEF2/ribosome-triggered GTP hydrolysis in vitro and slow down the peptide elongation of CPEB2-bound RNA in vivo [62]. Eukaryotic initiation factor 2 subunit 2 (eIF2S2), an initiation factor involved in mTOR pathway, is required in the initiation of protein translation and mediates the binding of tRNAmet to ribosome in a GTP-dependent manner. However, how the change of these mRNA abundance associated with dietary lysine would affect (inhibit or stimulate) transcription and translation warrants further investigation.

## 4. Materials and Methods

### 4.1. Animal Trial and Sample Collection

All the experimental protocols (as Project 13-051) involving caring, handling, and treatment of pigs were approved on 14-05-2014 by Mississippi State University Institutional Animal Care and Use Committee. A total of 9 crossbred (Large White × Landrace) late-stage finishing barrows (body weight 94.4 ± 6.7 kg) were housed in an environmentally controlled swine barn at the Leveck Animal Research Center, Mississippi State University. The pigs were randomly assigned to 9 individual feeding pens, and then assigned to one of the three dietary treatments according to a completely randomized experimental design. Each treatment group consisted of three pen replicates with one pig per pen.

A corn and soybean meal based diet (Diet 1; a lysine-deficient diet) was formulated to meet or exceed the NRC [1] recommended requirements for various nutrients including crude protein (CP) and essential AAs but not lysine. Diet 2 (a lysine-adequate diet) and Diet 3 (a lysine-excess diet) were formulated by adding l-lysine monohydrochloride (Archer Daniels Midland Co., Quincy, IL, USA) to Diet 1 at the expense of corn at rates of 0.35% and 0.70%, respectively (Table 3). The total lysine contents in Diets 1, 2, and 3 were 4.30, 7.10, and 9.80 g/kg (calculated, as-fed basis), respectively.

The feeding trial lasted five weeks, during which pigs were allowed ad libitum access to the experimental diets and fresh water. The pigs, feeders, waterers, and room temperature were checked 2–3 times (6:00 a.m. to 7:00 p.m.) on a daily basis. At the end of the feeding trial, the pigs were slaughtered in the Meat Science and Muscle Biology Laboratory of Mississippi State University, and muscle samples (approximately 5 g each) were collected from the middle portion of *longissimus dorsi* (between the 10th and 12th ribs) of each pig and snap frozen in liquid nitrogen immediately after the collection. The frozen muscle samples were then transferred to a −80 °C freezer for storage until laboratory analyses of gene expression.

### 4.2. Preparation of Total RNA

For each muscle sample, the total RNA was extracted from approximately 50 mg of frozen tissue using TRIzol Reagent (Invitrogen Corporation, Carlsbad, CA, USA) following the manufacturer’s instructions. Briefly, frozen tissue was homogenized in a 15 mL polypropylene centrifuge tube using a Polytron mixer (0.5 mL TRIzol per 50 mg tissue) and the homogenate transferred to 1.5 mL micro-centrifuge tubes. Chloroform (400 μL/tube) was used to separate RNA from DNA and proteins, and then the total RNA was precipitated with isopropyl alcohol (at 1:1 ratio) and washed with 750 μL of 75% ethanol. The resulting RNA was air-dried, dissolved in 60 μL RNase-free water, and stored at −80 °C freezer. The quality of the RNA samples was checked using an Agilent 2100 Bioanalyzer (Agilent Technologies, Santa Clara, CA, USA) and the results showed that all RNA samples had high quality with RNA integrity numbers (RIN) being from 8.2 to 9.3. The RNA concentrations were determined using a NanoDrop 1000 spectrophotometer (NanoDrop Technologies, Wilmington, DE, USA).

### 4.3. Microarray Analysis

The Affymetrix Porcine Gene 1.0 ST Array (Affymetrix, Inc., Santa Clara, CA, USA), which contains 394,589 probes representing 19,211 genes, was used to investigate the effect of dietary lysine on the potential change in the skeletal muscle gene expression of pigs. Microarray analysis was conducted at the University of Mississippi Medical Center Molecular and Genomics Core Facility (Jackson, MS, USA) according to the manufacturer’s recommended protocol (Affymetrix, Inc.). Briefly, the Ambion WT Expression Kit (Affymetrix, Inc.) was used to generate amplified sense-strand cDNA ready for fragmentation and labeling using the GeneChip WT Terminal Labeling and Controls Kit (Affymetrix, Inc.). Subsequently, the GeneChip Hybridization, Wash, and Stain Kit (Affymetrix, Inc.) was used to hybridize the gene chips in the GeneChip Hybridization Oven 640, using one chip per RNA sample. After hybridization, the chips were washed and stained on a GeneChip Fluidics Station 450. The reaction image and signals were read with a GeneChip Scanner (GCS 3000, 7G, Affymetrix, Inc.) and data were collected using the GCOS software (version 1.2, Affymetrix, Inc.). The raw gene expression intensity values from the software (i.e., the *.cel files) were imported into PGS software (Partek Inc., St. Louis, MO, USA) for analysis. For background correction, the algorithm of Robust Multichip Averaging adjusted with probe sequence and GC oligo contents was implemented. The background-corrected data were further converted into expression values using quantile normalization across all the chips and median polish summarization of multiple probes for each probe set [63,64].

All GeneChip transcripts were annotated using the NetAffx annotation database for Exon/Gene on Porcine GeneChip Array, provided online by the manufacturer (http://www.affymetrix.com/estore/analysis/index.affx; accessed 17 October 2015). The dietary treatment-induced effects on the expression of all the transcripts were subjected to one-way ANOVA analysis using the PGS software [65]. To achieve a higher degree of confidence, the transcripts showing treatment effects at the significance level of *p ≤* 0.01 were defined as being differentially expressed. These DEG were subjected to bioinformatic analysis, which used the canonical pathway analysis of the Core Analysis program of the IPA (IPA, 8.0-2602) online software (http://www.ingenuity.com; Ingenuity Systems, Inc., Redwood City, CA, USA).

### 4.4. Real-Time RT-PCR Analysis

Semi-quantitative real-time reverse-transcribed (RT)-PCR methodology was employed to verify the changes of the mRNA expression levels of nine selected genes, which were *PHGDH*, *PSPH*, *SCD*, *CIDEC*, *AMD1*, *ZIC1*, *ERLEC1*, *DNAJA1* and *ZNF181*, whose expression levels were found altered (*p ≤* 0.01) from the microarray analysis. These nine DEG were selected because they play critical roles in the metabolic pathways related to muscle growth and development.

First-strand cDNAs were reverse-transcribed from 1 µg of total RNA by using QuantiTect Reverse Transcription Kit (QIAGEN, Valencia, CA, USA). The semi-quantitative PCR analysis was performed using the Rotor-Gene Q System and the Rotor-Gene SYBR Green PCR Kit (QIAGEN), followed by melting curve analysis to verify the specificity and identity of the PCR products. The thermal cycling parameters were 95 °C for 5 min, followed by 40 cycles of 95 °C for 5 s and 60 °C for 10 s. Primers for the selected genes were designed by using PrimerQuest Tool (Integrated DNA Technologies, Coralville, IA, USA). The sequences of the designed primers and the relevant information associated with the PCR reactions for these selected genes are shown in Table 4. The endogenous control gene, *HPRT1* (hypoxanthine phosphoribosyltransferase), used for normalization of any variation during the process of sample preparation, is shown in Table 4 [66].

The ΔΔ*C*_t_ method was used for mRNA quantity calculation [67]. Briefly, the raw quantity of a given gene was normalized against the raw quantity of *HPRT1* reference gene of a given sample obtained from the Rotor-Gene Q System (QIAGEN, Valencia, CA, USA), and then the normalized level of the given gene of each sample was expressed as a quantity relative to the mean of the normalized quantities of the given gene of the Diet 2 treatment group.

### 4.5. Statistical Analysis

Dietary treatment effects on the relative mRNA expression levels, obtained from the microarray analysis for each gene transcript, were subjected to analysis of variance (ANOVA) for a completely randomized experimental design using the PGS software. For a higher degree of confidence, when the ANOVA test gave a *p* value less than or equal to 0.01, the treatment means were compared by two orthogonal contrasts: Diet 1 vs. Diet 2, and Diet 3 vs. Diet 2. Since this project was simply an animal nutrition study, no animals were sick and there were no clinical differences in terms of animal health status observed among the three treatments. Thus, few genes were expected to be altered by the lysine levels studied with respect to their mRNA expression. Therefore, the statistical procedure of False Discovery Rate that is usually used in animal disease models to control the false positives among the large numbers of significant positives was not employed in this study [63,68].

In addition, the dietary treatment effects on the relative expression levels of the selected DEG analyzed by real-time RT-PCR were also subjected to ANOVA and orthogonal contrast analyses (Diet 1 vs. Diet 2, and Diet 3 vs. Diet 2) using the General Linear Model procedure of SAS (SAS 9.4; SAS Institute Inc., Cary, NC, USA). When the contrast *p* values were less than or equal to 0.05, the comparison was considered to have a significant difference. For the canonical pathway analysis with IPA, the *p* values less than or equal to 0.05 or –log of *p*-value greater than or equal to 1.3 were considered significant.

## 5. Conclusions

Dietary lysine affected the expression levels of at least 131 gene transcripts in the *longissimus dorsi* muscle of the late-stage finishing pigs, of which 60 transcripts belonging to 59 genes were annotated in the NetAffx database. Dietary lysine deficiency may lead to: (1) increased muscle protein degradation via the ubiquitination pathway as indicated by the up-regulated *DNAJA1*, *HSP90AB1* and *UBE2B* mRNA levels; (2) reduced muscle protein synthesis via the upregulation of *RND3* and *ZIC1* mRNA levels; (3) increased serine and glycine synthesis in the muscle as indicated by the increased *PHGDH* and *PSPH* mRNA levels; and (4) increased lipid accumulation via the increased expression of *ME1*, *SCD*, and *CIDEC* mRNA levels. Dietary lysine excess may lead to: (1) decreased muscle protein degradation via the downregulation of mRNA levels of *DNAJA1*, *HSP90AA1*, *HSPH1*, and *UBE2D2*; and (2) reduced lipid biosynthesis via the decreased expression of *CFD* and *ME1* mRNA in porcine skeletal muscle. Collectively, dietary lysine may function as a signaling molecule to regulate protein turnover and lipid metabolism in the skeletal muscle of finishing pigs.

## Figures and Tables

**Table 1:** Genes differentially expressed in the skeletal muscle of finishing pigs fed a lysine-deficient vs. a lysine-adequate diet.

Gene Symbol	Gene Description	GenBank ^1^	*p*-Value ^2^
Down-regulated genes			
*CPEB2*	Cytoplasmic polyadenylation element binding protein 2	NM_001185049	<0.001
*CST9L*	Cystatin 9-like	XM_003134304	0.007
*ERLEC1*	Endoplasmic reticulum lectin 1	XM_003125147	0.011
*ESYT1*	Extended synaptotagmin-like protein 1	XM_003126262	0.009
*HOXA11B*	Homeobox A11	AF453292	0.001
*LCLAT1*	Lysocardiolipin acyltransferase 1	NM_001142845	0.032
*MYL6*	Myosin, light chain 6	NM_001163997	0.011
*RASSF7*	Ras association domain family member 7	XM_003122393	0.004
*UBTD1*	Ubiquitin domain containing 1	XM_003359315	0.003
*ZMAT5*	Zinc finger, matrin-type 5	XM_001929010	0.035
*ZNF181*	Zinc finger protein 181	NM_001244818	0.005
Up-regulated genes			
*AMD1*	Adenosylmethionine decarboxylase 1	XM_003121345	0.014
*ATP6V1G2*	Atpase, H^+^ transporting, lysosomal 13 kDa, V1 subunit G2	NM_001145380	0.002
*C7H6orf136*	Chromosome 6 open reading frame 136	NM_001243459	0.003
*CCDC25*	Coiled-coil domain containing 25	NM_001243572	0.023
*CD164*	CD164 molecule	XM_001924626	0.022
*CHIT1*	Chitinase 1	XM_003130296	0.005
*CHORDC1*	Cysteine and histidine-rich domain (CHORD) containing 1	NM_001113446	0.010
*CHSY3*	Chondroitin sulfate synthase 3	XM_003123906	0.049
*CIDEC*	Cell death-inducing DFFA-like effector c	NM_001112689	0.024
*DNAJA1*	Dnaj (Hsp40) homolog, subfamily A, member 1	NM_001244163	0.003
*eIF2S2*	Eukaryotic translation initiation factor 2 subunit 2	XM_005672861	0.002
*FUT1*	Fucosyltransferase 1	NM_214068	0.003
*GABPB1*	GA binding protein transcription factor, β subunit 1	XM_005659610	0.002
*GPR182*	G protein-coupled receptor 182	XM_003126290	0.000
*H1FOO*	Oocyte-specific H1 histone	NM_001205063	0.016
*HOXA4*	Homeobox A4	XM_003134841	0.037
*HSP90AB1*	Heat shock protein 90 kDa α (cytosolic), class B member 1	NM_001244433	0.007
*LGALS13*	Lectin, galactoside-binding, soluble, 13	NM_001142841	0.003
*ME1*	Malic enzyme 1, NADP^+^-dependent, cytosolic	XM_001924333	0.050
*MIR3187*	MicroRNA 3187	NR_036154	0.002
*PHGDH*	Phosphoglycerate dehydrogenase	NM_001123162	0.000
*PSPH*	Phosphoserine phosphatase	NM_001243221	0.009
*RND3*	Rho family GTPase 3	NM_214296	0.003
*SCD*	Stearoyl-CoA desaturase (δ-9-desaturase)	NM_213781	0.023
*SERP1*	Stress-associated endoplasmic reticulum protein 1	NM_001243260	0.039
*TXNL1*	Thioredoxin-like 1	NM_001244276	0.030
*UBE2B*	Ubiquitin-conjugating enzyme E2B	NM_001257356	0.011
*ZIC1*	Zic family member 1	XM_003358599	0.011

^1^ The accession number of the gene sequence resided in the GenBank database (http://www.ncbi.nlm.nih.gov); and ^2^ *p*-Value was obtained from the orthogonal contrast analysis using the Partek Genomics Suite software (Partek Inc., St. Louis, MO, USA). A change in gene mRNA abundance was considered significant when the *p*-value was less than or equal to 0.05.

**Table 2:** Genes differentially expressed in the skeletal muscle of finishing pigs fed a lysine-excess vs. a lysine-adequate diet.

Gene Symbol	Gene Description	GenBank ^1^	*p*-Value ^2^
Down-regulated genes			
*ALKBH7*	AlkB, alkylation repair homolog 7	XM_003123112	0.001
*AMD1*	Adenosylmethionine decarboxylase 1	XM_003121345	0.013
*CFAP20*	Cilia and flagella associated protein 20	NM_001244786	0.001
*CFD*	Complement factor D (adipsin)	XM_003122985	0.009
*CHORDC1*	Cysteine and histidine-rich domain (CHORD) containing 1	NM_001113446	0.006
*CLCA2*	Chloride channel accessory 2	XM_003125930	0.003
*CNTFR*	Ciliary neurotrophic factor receptor	XM_003130672	0.027
*DNAJA1*	Dnaj (Hsp40) homolog, subfamily A, member 1	NM_001244163	0.004
*ERLEC1*	Endoplasmic reticulum lectin 1	XM_003125147	0.004
*HOXA4*	Homeobox A4	XM_003134841	0.014
*HSP90AA1*	Heat shock protein 90 kDa α (cytosolic), class A member 1	NM_213973	0.034
*HSPH1*	Heat shock 105 kDa/110 kDa protein 1	NM_001097504	0.014
*LCLAT1*	Lysocardiolipin acyltransferase 1	NM_001142845	0.001
*ME1*	Malic enzyme 1, NADP^+^-dependent, cytosolic	XM_001924333	0.027
*MFAP3*	Microfibrillar-associated protein 3	XM_003134126	0.001
*MORF4L2*	Mortality factor 4 like 2	XM_003135267	0.033
*SERP1*	Stress-associated endoplasmic reticulum protein 1	NM_001243260	0.016
*SLC9A2*	Solute carrier family 9, subfamily A (NHE2, cation proton antiporter 2), member 2	NM_001100189	0.010
*TMCO6*	Transmembrane and coiled-coil domains 6	XM_003124040	0.003
*UBE2D2*	Ubiquitin-conjugating enzyme E2D 2	NM_001078673	0.028
*UBTD1*	Ubiquitin domain containing 1	XM_003359315	0.009
*ZMAT5*	Zinc finger, matrin-type 5	XM_001929010	0.001
Up-regulated genes			
*CHIT1*	Chitinase 1 (chitotriosidase)	XM_003130296	0.009
*CHSY3*	Chondroitin sulfate synthase 3	XM_003123906	0.001
*CNTROB*	Centrobin, centrosomal BRCA2 interacting protein	XM_003358269	0.007
*CXCR6*	Chemokine (C-X-C motif) receptor 6	NM_001001623	0.000
*DBX1*	Developing brain homeobox 1	XM_003122916	0.002
*DND1*	DND microrna-mediated repression inhibitor 1	XM_003124043	0.009
*GPR182*	G protein-coupled receptor 182	XM_003126290	0.008
*H1FOO*	Oocyte-specific H1 histone	NM_001205063	0.003
*LGALS13*	Lectin, galactoside-binding, soluble, 13	NM_001142841	0.005
*MYO5B*	Myosin VB	XM_003121434	0.001
*PDCL*	Phosducin-like	XM_001927696	0.003
*XKR4*	XK, Kell blood group complex subunit-related family, member 4	XM_003355057	0.008
*ZNF181*	Zinc finger protein 181	NM_001244818	0.037

^1^ The accession number of the gene sequence resided in the GenBank database (http://www.ncbi.nlm.nih.gov); ^2^ *p*-Value was obtained from the orthogonal contrast analysis using the Partek Genomics Suite software (Partek Inc., St. Louis, MO, USA). A change in gene mRNA abundance was considered significant when the *p*-value was less than or equal to 0.05.

**Table 3:** Feed ingredients and nutrient composition of experimental diets fed to finishing pigs (as-fed basis) ^1^.

Item	Diet 1	Diet 2	Diet 3
Ingredients (g/kg)			
Corn	908.44	904.94	901.44
Soybean meal	64.00	64.00	64.00
Canola oil	8.00	8.00	8.00
l-Lysine-HCl (98.5%)	0.00	3.50	7.00
dl-Methionine (99.0%)	0.40	0.40	0.40
l-Threonine (98.5%)	0.90	0.90	0.90
l-Tryptophan (99.0%)	0.35	0.35	0.35
Limestone	6.50	6.50	6.50
Dicalcium phosphate	9.00	9.00	9.00
Salt	2.00	2.00	2.00
Mineral premix ^2^	0.33	0.33	0.33
Vitamin premix ^3^	0.08	0.08	0.08
Total	1000.0	1000.0	1000.0
Composition (g/kg) ^4^			
Metabolizable energy (kcal/kg)	3319	3323	3326
Crude protein	104.5	107.5	110.5
Total lysine	4.33	7.08	9.82
Total methionine	2.37	2.36	2.36
Total threonine	5.02	5.01	5.00
Total tryptophan	1.40	1.40	1.39
Total Ca	4.58	4.58	4.58
Total P	4.32	4.31	4.30

^1^ Diets 1, 2, and 3 were formulated to contain total lysine 4.33, 7.08, and 9.82 g/kg (as-fed basis), respectively, of which Diets 2 and 3 were formulated by adding 3.50 and 7.00 g/kg l-lysine-HCl (Archer Daniels Midland Co., Quincy, IL, USA) to Diet 1 at the expense of corn; ^2^ The mineral premix contained Ca 132 g/kg, Cu 10.0 g/kg, Fe 80.0 g/kg, Mn 50.0 g/kg, Zn 100.0 g/kg, I 500 mg/kg, and Se 300 mg/kg; ^3^ Each kilogram of vitamin premix contained the following: 22.05 million IU vitamin A, 3.31 million IU vitamin D3, 66,138 IU vitamin E, 88 mg vitamin B12, 220 mg biotin, 8,818 mg menadione, 15,432 mg riboflavin, 61,728 mg d-pantothenic acid, and 88,183 mg niacin and ^4^ Calculated major nutrients.

**Table 4:** The primer pairs used in the semi-quantitative real-time RT-PCR analysis of the selected genes.

Gene Symbol ^1^	GenBank ^2^	Sequence (5′–3′) ^3^	Amplicon Size (bp)
*PHGDH*	NM_001123162	F: GCGGTTTGGTTTAGGTGTTTC	113
		R: AAGGGTCCAGGCTATCACT	
*PSPH*	NM_001243221	F: CTGCAGGCTCCAGTTTAGTT	97
		R: CTCGCAGAGTCTTTACCAACA	
*SCD*	NM_213781	F: CCCAAGGCAGACAAGAGAATAG	91
		R: GTGTTGACGACTGAGGTTACAG	
*CIDEC*	NM_001112689	F: CCAACTCTCCCTCTCCCATAA	106
		R: CATGTTCAGGCAACCAATGAAG	
*AMD1*	XM_003121345	F: TCCACAAGTCAAGTCCTCTAATG	108
		R: CCATGGAGAGGAACGAATCAA	
*ZIC1*	XM_003358599	F: CGACCGACGCTTTGCTAATA	97
		R: GTAGGACTTGTCGCACATCTT	
*ERLEC1*	XM_003125147	F: GCTGGCTATCCTTTGTACTCTC	109
		R: CAACACTGCTTGTGGACATTT	
*DNAJA1*	NM_001244163	F: GGTGGTAAGAAAGGAGCAGTAG	93
		R: CTGAACCATTCCAGGTCCTATT	
*ZNF181*	NM_001244818	F: GCCTTCAGCCAAAGCAAATC	85
		R: AGGCTTTCCCACATTCACTAC	
*HPRT1* ^4^	NM_001032376	F: GCTATGCCCTTGACTACAATGA	102
		R: TTGAACTCTCCTCTTAGGCTTTG	

^1^ *PHGDH* = Phosphoglycerate dehydrogenase; *PSPH* = Phosphoserine phosphatase; *SCD* = Stearoyl-CoA desaturase; *CIDEC* = Cell death-inducing DFFA-like effector c; *AMD1* = Adenosylmethionine decarboxylase 1; *ZIC1* = Zic family member 1; *ERLEC1* = Endoplasmic reticulum lectin 1; *DNAJA1* = DnaJ (Hsp40) homolog, subfamily A, member 1; *ZNF181* = Zinc finger protein 181; *HPRT1* = Hypoxanthine phosphoribosyltransferase 1; ^2^ The accession number of the cDNA sequences retrieved from the GenBank database (http://www.ncbi.nlm.nih.gov) for primer design; ^3^ F = a forward primer, and R = a reverse primer; and ^4^ *HPRT1* was selected as an internal control reference gene for the purpose of normalization of the expression of other gene.

## Supplementary Materials

Supplementary materials can be found at www.mdpi.com/1422-0067/18/4/885/s1.

## Abbreviations

AA	Amino acid
ADG	Average daily gain
Akt	Protein Kinase B
ANOVA	Analysis of variance
ARS	Agricultural Research Service
CP	Crude protein
CT	Threshold cycle
DEG	Differentially expressed genes or gene transcripts
ER	Endoplasmic reticulum
GC	Guanine-cytosine
GEO	Gene Expression Omnibus
GCOS	GeneChip Operating Software
IPA	Ingenuity Pathways Analysis
MAPK	Mitogen-activated protein kinase
MIAMIE	Minimum information about a microarray experiment
NIFA	National Institute of Food and Agriculture
NRC	National Research Council
PGS	Partek Genomics Suite
PI3K	Phosphatidylinositol 3-kinase
PM	Plasma membrane
PTEN	Phosphatase and tensin homolog
RT	Reverse transcription
SAS	Statistical Analysis System
USDA	U.S. Department of Agriculture
