# Supplementary material for: A Systems Biology Approach Using Transcriptomic Data Reveals Genes and Pathways in Porcine Skeletal Muscle Affected by Dietary Lysine
Source: Int J Mol Sci. 2017 Apr 21;18(4):885. doi: 10.3390/ijms18040885 (PMC5412465; doi:10.3390/ijms18040885)
Supplement: Supplementary file 1 [file ijms-18-00885-s001.pdf]

Supplemental Table S1 A list of genes or gene transcripts that were differentially expressed among pigs fed three different diets

Transcript ID[a]	Gene Symbol	Gene Description[b]	GenBank[c]	*P* -value[d]
15263546	ALKBH7	AlkB, alkylation repair homolog 7	XM_003123112	0.002
15184804	AMD1	Adenosylmethionine decarboxylase 1	XM_003121345	0.001
15314841	ATP6V1G2	Atpase, H+ transporting, lysosomal 13 kda, V1 subunit G2	NM_001145380	0.003
15314665	C7H6orf136	Chromosome 6 open reading frame 136	NM_001243459	0.009
15229626	CCDC25	Coiled-coil domain containing 25	NM_001243572	0.006
15184906	CD164	CD164 molecule	XM_001924626	0.005
15296214	CFAP20	Cilia and flagella associated protein 20	NM_001244786	0.002
15255767	CFD	Complement factor D (adipsin)	XM_003122985	0.003
15332101	CHIT1	Chitinase 1 (chitotriosidase)	XM_003130296	0.010
15329843	CHORDC1	Cysteine and histidine-rich domain (CHORD) containing 1	NM_001113446	0.001
15259346	CHSY3	Chondroitin sulfate synthase 3	XM_003123906	0.002
15220865	CIDEC	Cell death-inducing DFFA-like effector c	NM_001112689	0.006
15286963	CLCA2	Chloride channel accessory 2	XM_003125930	0.010
15202461	CNTFR	Ciliary neurotrophic factor receptor	XM_003130672	0.008
15209728	CNTROB	Centrobin, centrosomal BRCA2 interacting protein	XM_003358269	0.006
15319886	CPEB2	Cytoplasmic polyadenylation element binding protein 2	NM_001185049	0.001
15248533	CST9L	Cystatin-9 like	XM_003134304	0.008
15215166	CXCR6	Chemokine (C-X-C motif) receptor 6	NM_001001623	0.001
15255294	DBX1	Developing brain homeobox 1	XM_003122916	0.004
15202526	DNAJA1	Dnaj (Hsp40) homolog, subfamily A, member 1	NM_001244163	0.000
15267072	DND1	DND microrna-mediated repression inhibitor 1	XM_003124043	0.004
15246704	EIF2	Eukaryotic translation initiation factor 2	XM_005672861	0.002
15249075	EIF2	Eukaryotic translation initiation factor 2	XM_005672861	0.002
15276460	ERLEC1	Endoplasmic reticulum lectin 1	XM_003125147	0.009
15288283	ESYT1	Extended synaptotagmin-like protein 1	XM_003126262	0.006
15297540	FUT1	Fucosyltransferase 1	NM_214068	0.004
15194689	GABPB1	GA binding protein transcription factor, beta subunit 1	XM_005659610	0.005
15288427	GPR182	G protein-coupled receptor 182	XM_003126290	0.001
15216623	H1FOO	Oocyte-specific H1 histone	NM_001205063	0.007
15251282	HOXA11B	Homeobox A11	AF453292	0.001
15251306	HOXA4	Homeobox A4	XM_003134841	0.003
15313557	HSP90AA1	Heat shock protein 90kda alpha (cytosolic), class A member 1	NM_213973	0.009
15310151	HSP90AB1	Heat shock protein 90kda alpha (cytosolic), class B member 1	NM_001244433	0.005

15204824	HSPH1	Heat shock 105kda/110kda protein 1	NM_001097504	0.008
15276892	LCLAT1	Lysocardiolipin acyltransferase 1	NM_001142845	0.003
15302843	LGALS13	Lectin, galactoside-binding, soluble, 13	NM_001142841	0.005
15253268	LOC100625033	Uncharacterized	XM_003360198	0.009
15185014	ME1	Malic enzyme 1, $NADP^+$-dependent, cytosolic	XM_001924333	0.005
15245225	MFAP3	Microfibrillar-associated protein 3	XM_003134126	0.003
15263058	MIR3187	Microrna 3187	NR_036154	0.006
15337580	MORF4L2	Mortality factor 4 like 2	XM_003135267	0.010
15292502	MYL6	Myosin, light chain 6	NM_001163997	0.007
15194051	MYO5B	Myosin VB	XM_003121434	0.001
15198805	PDCL	Phosducin-like	XM_001927696	0.003
15286002	PHGDH	Phosphoglycerate dehydrogenase	NM_001123162	0.000
15268536	PSPH	Phosphoserine phosphatase	NM_001243221	0.006
15260400	RASSF7	Ras association domain family member 7	XM_003122393	0.004
15235102	RND3	Rho family gtpase 3	NM_214296	0.007
15228053	SCD	Stearoyl-coa desaturase (delta-9-desaturase)	NM_213781	0.007
15221605	SERP1	Stress-associated endoplasmic reticulum protein 1	NM_001243260	0.003
15270271	SLC9A2	Solute carrier family 9, subfamily A (NHE2, cation proton antiporter 2), member 2	NM_001100189	0.005
15259768	TMCO6	Transmembrane and coiled-coil domains 6	XM_003124040	0.004
15194165	TXNL1	Thioredoxin-like 1	NM_001244276	0.007
15218159	UBE2B	Ubiquitin-conjugating enzyme E2B	NM_001257356	0.010
15321630	UBE2D3	Ubiquitin-conjugating enzyme E2D 3	NM_001078673	0.006
15227915	UBTD1	Ubiquitin domain containing 1	XM_003359315	0.007
15284156	XKR4	XK, Kell blood group complex subunit-related family, member 4	XM_003355057	0.006
15221539	ZIC1	Zic family member 1	XM_003358599	0.005
15225194	ZMAT5	Zinc finger, matrin-type 5	XM_001929010	0.004
15302378	ZNF181	Zinc finger protein 181	NM_001244818	0.001
15344815		---	---	0.000
15345937		---	---	0.000
15349723		---	---	0.000
15347387		---	---	0.001
15182061		---	---	0.001
15258761		---	---	0.001
15233088		---	---	0.001
15240879		---	---	0.002
15332195		---	---	0.002

15276173	---	---	0.002
15180499	---	---	0.002
15232252	---	---	0.002
15195023	---	---	0.002
15272119	---	---	0.002
15342067	---	---	0.002
15346419	---	---	0.003
15351163	---	---	0.003
15209896	---	---	0.003
15181739	---	---	0.003
15344923	---	---	0.003
15290393	---	---	0.003
15341709	---	---	0.003
15181633	---	---	0.004
15342887	---	---	0.004
15279019	---	---	0.004
15182949	---	---	0.004
15347493	---	---	0.004
15181475	---	---	0.005
15182773	---	---	0.005
15345617	---	---	0.005
15256666	---	---	0.005
15347037	---	---	0.005
15339967	---	---	0.005
15340953	---	---	0.006
15182761	---	---	0.006
15350707	---	---	0.006
15350863	---	---	0.006
15184164	---	---	0.006
15282393	---	---	0.006
15286988	---	---	0.006
15273431	---	---	0.006
15280254	---	---	0.007
15339819	---	---	0.007
15351493	---	---	0.007
15314718	---	---	0.007

15341149	---	---	0.007
15286729	---	---	0.007
15326843	---	---	0.007
15332776	---	---	0.007
15349625	---	---	0.008
15349601	---	---	0.008
15182561	---	---	0.008
15249513	---	---	0.008
15347693	---	---	0.008
15221666	---	---	0.008
15182789	---	---	0.008
15344477	---	---	0.008
15244806	---	---	0.008
15349439	---	---	0.008
15346091	---	---	0.009
15340753	---	---	0.009
15182951	---	---	0.009
15339581	---	---	0.009
15281243	---	---	0.009
15245843	---	---	0.009
15339337	---	---	0.009
15271887	---	---	0.010
15279096	---	---	0.010
15182455	---	---	0.010
15342033	---	---	0.010
15253611	---	---	0.010

[a] The gene transcript ID were retrived from the NetAffx calssification system (Affymetrix, Inc.).

[b] The "---" signs indicate that no annotation had been made to the corresponding DNA or RNA sequences of porcine origin.

[c] The accession numbesr for the gene sequences resided in the GenBank database (http://www.ncbi.nlm.nih.gov).

[d] *P* -value was obtained from the ANOVA test using the Partek Genomics Suite (PGS) software.
